# Early cup migration and wear as predictors for later aseptic loosening: a secondary evaluation of a randomized controlled RSA trial on cemented hip arthroplasties with 18-year follow-up

**DOI:** 10.2340/17453674.2025.44328

**Published:** 2025-08-15

**Authors:** Håkon Greve JOHANNESSEN, Geir HALLAN, Thomas KADAR, Stein Atle LIE, Stein Håkon Låstad LYGRE, Anne Marie FENSTAD, Kristin HAUGAN, Paul Johan HØL, Mona BADAWY, Benedikt JONSSON, Kari INDREKVAM, Arild AAMODT, Ove FURNES

**Affiliations:** 1 Department of Clinical Medicine, Faculty of Medicine, University of Bergen, Bergen, Norway; 2 The Norwegian Arthroplasty Register, Department of Orthopaedic Surgery, Haukeland University Hospital, Bergen, Norway; 3 Coastal Hospital in Hagavik, Department of Orthopaedic Surgery, Haukeland University Hospital, Bergen, Norway; 4 Department of Physical Medicine and Rehabilitation, Haukeland University Hospital, Bergen, Norway; 5 Centre for Translational Oral Research (TOR), Department of Clinical Dentistry, Faculty of Medicine, University of Bergen, Norway; 6 Department of Occupational Medicine, Haukeland University Hospital, Bergen, Norway; 7 Department of Orthopaedic Surgery, St. Olavs Hospital, Trondheim, Norway; 8 Department of Orthopaedic Surgery, Biomatlab, Haukeland University Hospital, Bergen, Norway; 9 Landshospitali University Hospital, Reykjavik, Iceland; 10 Department of Orthopaedic Surgery, Lovisenberg Diakonal Hospital, Oslo, Norway

## Abstract

**Background and purpose:**

There is no clear evidence on whether migration or wear is the best predictor for later acetabular cup loosening. We aimed to investigate whether early wear or migration, measured via radiostereometric analysis (RSA), predicts later cup loosening. We also compared long-term aseptic loosening rates between conventional (CPE) and highly crosslinked polyethylene (XLPE) cups.

**Methods:**

Data was drawn from a randomized controlled trial (RCT) (ClinicalTrials.Gov NCT00698672) of 150 patients receiving cemented total hip arthroplasties (THAs), with 10-year RSA follow-up. 5 groups were assessed based on implant combinations (Charnley or Spectron EF stems with CPE or XLPE cups and CoCr or Oxinium heads). Migration and wear up to 2 years were evaluated against 18-year cup survival using receiver operating characteristic (ROC) curves.

**Results:**

19 cups (17 CPE, 2 XLPE) were loose at final follow-up. The area under the ROC curve (AUC) was 0.56 (95% confidence interval [CI] 0.40–0.73) for early migration and 0.85 (CI 0.77–0.94) for early polyethylene (PE) wear, with a difference of 0.29 (CI 0.09–0.49). Hazard ratio for loosening was 0.88 (CI 0.20–3.89) for early migration > 0.2 mm and 19.4 (CI 2.55–147) for early wear > 0.2 mm. At 18 years, survival free of aseptic loosening was 65% (CI 48–77) for CPE and 96% (CI 85–99) for XLPE cups, with a 9-fold higher risk of loosening for CPE.

**Conclusion:**

Early polyethylene wear, not migration, predicted long-term cup loosening. XLPE showed superior long-term performance over CPE with less wear, cup loosening, and revision.

Radiostereometric analysis (RSA) is a method that can be used for in vivo measurements of movement of implants. Because of the high precision and prospective value of this method, few patients are at risk in the evaluation of hip or knee prostheses [1,2]. In total hip arthroplasty (THA), early cup migration has been found to be an indicator for the risk of implant loosening at 10 years, with different thresholds introduced for varying levels of risk of an implant based on RSA at 2 years [3]. Precise measurements can also be done of the wear (penetration) of polyethylene (PE) [4]. Wear under 0.1 mm/year has been regarded as a threshold where osteolysis and subsequent loosening is rarely observed [5]. However, a dose–response mechanism has also been proposed, with an increased occurrence of osteolysis with increasing wear rate [6]. It has been proposed that conducting RSA studies within 2-year follow-up is sufficient for evaluating the migration and wear patterns of an implant, and hence predicting its long-term performance [7]. However, more long-term RSA studies are required to evaluate migration patterns over time [8]. There is, though, no clear evidence as to whether migration or wear is the best predictor for later acetabular cup loosening [4,8].

Our earlier studies with up to 10-year data showed a significant reduction in both PE wear and proximal cup migration with highly crosslinked polyethylene (XLPE) compared with conventional (CPE) in THA of otherwise identical design.

Our primary aim was to compare the predictive value of early acetabular cup PE wear versus migration, as measured by radiostereometric analysis (RSA), in determining long-term cup loosening in THA. Secondary aims were to evaluate previously suggested thresholds for cup migration and PE wear within the context of this cohort and provide sensitivity and specificity of these thresholds and evaluate the survival of CPE versus XLPE cups with up to 18-year follow-up, with aseptic loosening as the main endpoint.

## Methods

This study is an observational follow-up of a cohort from an RCT with 18-year follow-up. The paper is written according to the STROBE guidelines for observational studies [9].

We used data from a study of 5 different cemented THAs which utilized CPE and XLPE articulating with CoCr or Oxinium heads, as well as a group with Charnley prostheses, with results published for 2-, 5-, and 10-year follow-up [10-12].

From November 2004 to June 2007, 150 patients (70% female) with a mean age of 70 years (range 59–80) were enrolled to undergo THA for the treatment of primary or secondary hip osteoarthritis. The methodology and results of the RSA measurements have been published previously for the 2-, 5-, and 10-year follow-up periods [10-12]. Informed consent was obtained from all participants prior to their inclusion in the study. For patients with bilateral hip osteoarthritis, only 1 hip was selected for inclusion. Exclusion criteria included a BMI greater than 35, uncontrolled cardiopulmonary conditions, malignancies, dementia, rheumatoid arthritis, or any other significant systemic disorders.

**Figure 1:**
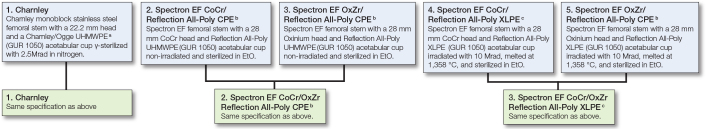
Flowchart demonstrating the 5 originally randomized groups with their articulations, and the merging into 3 groups for survival analysis. EtO: ethylene oxide. **^a^** UHMWPE: ultra-high molecular weight polyethylene. **^b^** CPE: conventional polyethylene. **^c^** XLPE: highly crosslinked polyethylene.

Originally, the patients were randomly assigned to 1 of 5 groups, 1 Charnley/Ogee group and 4 Spectron EF/Reflection All-Poly groups, utilizing both CoCr and Oxinium femoral heads, as well as CPE and XLPE acetabular liners (Figure 1) [10-12].

Patients were operated on using a modified direct lateral approach, in the lateral decubitus position, in an operating theater with laminar airflow, under spinal anesthesia [13]. The acetabulum was reamed to bleeding subchondral bone. The components were inserted with Palacos R with gentamicin cement (Schering-Plough, Labo N.V., Heist-Op-Den-Berg, Belgium) using a third-generation cementing technique. Femoral stem insertion was performed 5 minutes after cement mixing; cup insertion 6 minutes after mixing. Patients received tranexamic acid before surgery, perioperative systemic antibiotics (4 doses of cefuroxime 2 g or 2 doses of clindamycin 0.6 g in the presence of penicillin allergy) and low molecular-weight heparin (dalteparin 5000 IE sc) for 5 weeks. Patients were allowed partial weightbearing with crutches from the 1st postoperative day, until 6 weeks postoperatively.

### Outcome measures

The median time for the postoperative RSA examination was 11 days (range 9–15 days) following surgery, with additional follow-up examinations conducted at 3, 6, 12, 60, and 120 months post-surgery. All imaging was performed by the same radiographer. The RSA method was standardized according to the established guidelines at the time [10,14]. The acetabular cups came with manufacturer-embedded tantalum markers: 10 × 0.8 mm for Charnley, 6 × 1.0 mm for Reflection. During surgery, 6–9 additional markers were placed in the periprosthetic pelvic bone: 1 mm for the Charnley group and 0.8 mm for the Reflection groups. A uniplanar technique was used with the calibration cage (Cage 43, RSA Biomedical, Umeå, Sweden) placed beneath the examination table. Patients were examined in the supine position. Simultaneous exposures were taken using a gantry-mounted and a portable X-ray tube. Imaging was performed with high-definition digital plates (Agfa, Mortsel, Belgium CR MD 4.0), and readings were processed using the ADC compact digitizer (Agfa HealthCare, Mortsel, Belgium). To assess cup penetration, the movement of the femoral head center, represented as a point, was tracked using the tantalum markers in the polyethylene (PE) liner as a fixed reference. Cup migration was determined by tracking the movement of the rigid body created by the markers in the PE, with the periprosthetic bone serving as the reference. The penetration, translation, and rotation of the cup were calculated along the horizontal (X), longitudinal (Y), and sagittal (Z) axes, based on signed values, using the UmRSA Digital Measure software, version 5.0 (RSA Biomedical, Umeå, Sweden).

The proximal cup penetration of the femoral head (y-translation) at 2-year RSA follow-up was used to define early PE wear, as the amount of bedding-in was similar for all groups. Proximal migration along the y-axis at 2 years was used to define early cup migration. We also evaluated stabilization of the cups, defined as the delta migration between 1- and 2-year RSA measurements. ROC curves were created based on these variables, and the main outcome was AUC for early migration and PE wear. An AUC of 0.5 is the equivalent of tossing a coin as a diagnostic test. Suggestions have been made to evaluate the AUC regarding a value between 0.5 and 0.7 as poor, between 0.7 and 0.8 as good, 0.8 and 0.9 very good, and above 0.9 as excellent [15]. The threshold of 0.2 mm of proximal cup migration, which has previously been established for group-level analyses, and a wear rate of 0.1 mm/year were defined as previously suggested thresholds, and were evaluated for their predictive value [3,5]. For early PE wear 0.2 mm was chosen as the threshold, from a wear rate of 0.1 mm/year, as bedding-in was similar across the different THAs.

Information regarding implant revisions was collected from the Norwegian Arthroplasty Register (NAR), patient files, and radiographs. NAR has 97% completeness of reporting of primary hip arthroplasties and 91% for revisions [16]. The follow-up was extended till December 31, 2022. The latest available radiographs taken before this date were examined for radiological signs of implant loosening. Specifically, a radiolucent line exceeding 1 mm in all 3 DeLee & Charnley zones, or clear evidence of implant migration were regarded as signs of loosening [17]. The first 30 images were examined by the first author in cooperation with an experienced orthopedic surgeon (GH), and the rest by the first author alone. All cases that were considered as loose were verified by the experienced surgeon, as well as cases of doubt. In our survival analysis “loose” cups were defined as cups revised due to loosening, and those radiographically determined to be loose.

### Statistics

**Figure 2:**
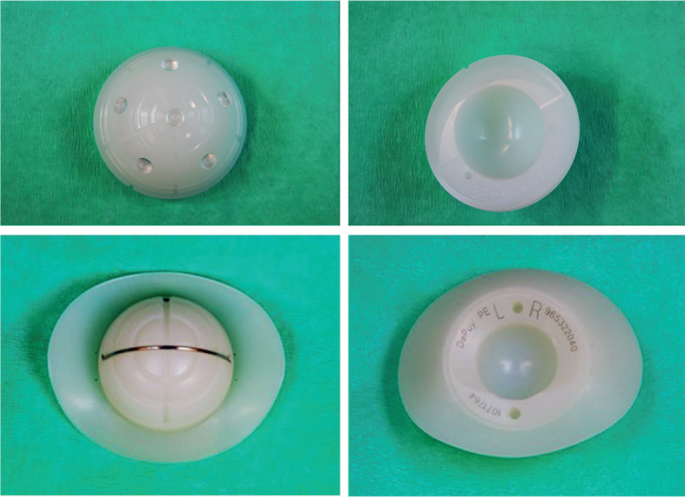
Images of the 2 cup designs used in the study. Reflection All-Poly (top); Charnley/Ogee cup (bottom).

After a power analysis, a group size of 30 individuals was chosen for the initial RSA evaluation of wear and migration [10,11]. In evaluation of early migration and PE wear as predictors for later loosening, all Spectron EF/Reflection All-Poly THAs were merged into 1 group. The Charnley group was not included in the main analysis due to being of a different design from the Spectron EF/Reflection All-Poly groups (Figure 2). Analyses that includes Charnley cups are provided in the Supplementary data. Receiver operating characteristic (ROC) curves were created based on loosening or not at 18-year follow up, with respect to early migration and PE wear [18]. In addition, a third curve, combining the effects of both migration and wear, from a logistic regression, was added as a visual aid. Area under the ROC curve (AUC) is reported. Revision due to aseptic or radiographic loosening was defined as the event for the ROC curves. AUC was calculated and compared, using DeLong’s test, for both early migration and PE wear [19]. Furthermore, Kaplan–Meier survival curves were created for time to loosening, stratified by early wear and migration. Hazard ratios (HR) for loosening were created using Cox regression, for both early wear and migration over 0.2 mm [12].

The survival analysis merged the Spectron EF/Reflection All-Poly groups into the 2 types of PE (CPE and XLPE) as there was no difference in wear or migration at 2 years between the 2 head materials (CoCr and Oxinium) [10]. Kaplan–Meier survival probabilities with log rank tests were calculated to compare survival probabilities for CPE and XLPE Reflection cups. Unadjusted Cox regressions were used to compare the risk of loosening between CPE and XLPE cups. Events were recorded at the date of revision due to aseptic loosening or the earliest radiograph with definite signs of loosening. Patient death or revisions for other reasons were censored data points. All tests were 2-sided, and the significance level was set to 0.05. Statistics were compiled using SPSS version 29 (IBM Corp, Armonk, NY, USA), and STATA version 18 (StataCorp, College Station, TX, USA). The R 4.2.2 statistical software package (R Foundation for Statistical Computing, Vienna, Austria) was used for creating the figures.

### Ethics, registration, data sharing, funding, and disclosures

The main RSA study was registered in ClinicalTrials.Gov (NCT00698672) and approved by the Western Norway regional ethics committee (REK number 2014-02370). The primary aim was reported at 2 years, and the final 10-year RSA-follow-up for the acetabular cups has recently been published [10-12]. De-identified data may be shared upon request. ChatGPT (OpenAI, San Francisco, CA, USA) was used to reformulate sections of the text. The study was jointly financed by OrtoMedic AS, Smith & Nephew Norway AS, and the Regional Health Board of Western Norway. Complete disclosure of interest forms according to ICMJE are available on the article page, doi: 10.2340/17453674.2025.44328

## Results

**Figure 3:**
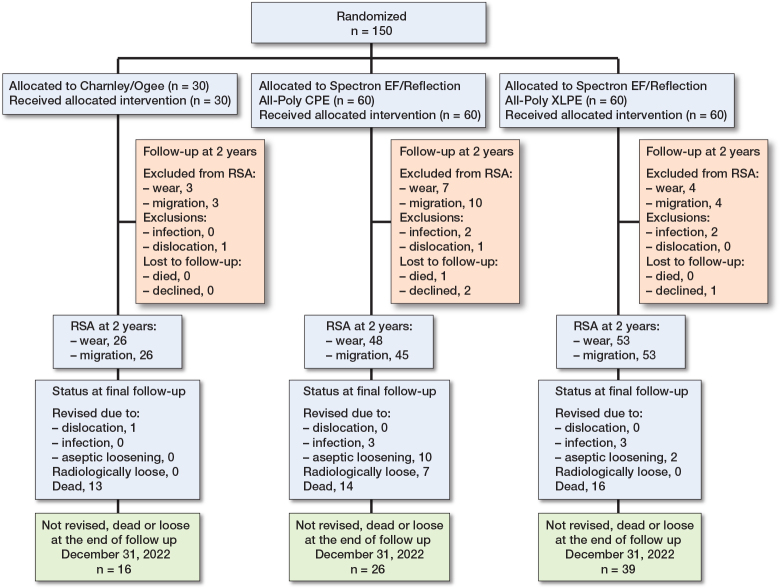
Flowchart for the RSA at 2 years (and Kaplan–Meier survival analysis until 18 years’ follow-up) in cemented total hip arthroplasty in 3 different groups. Endpoint: Aseptic loosening of cup (radiological or surgical revision) with follow-up until end of December 2022.

At the end of December 2022, a total of 81 cups were considered well fixed, 19 had undergone revision for aseptic loosening or were radiologically loose, and 50 were censored due to patient death or revision for reasons other than aseptic loosening (Figure 3). During the 18-year study period, 43 patients died and 7 were revised for reasons other than aseptic loosening.

There were no differences in demographic characteristics between the groups at baseline, other than cup size due to the Charnley Ogee cup only being used in smaller sizes, 40 and 43 mm (Table).

**Table:** Cemented total hip arthroplasty with 3 different groups: demographics of the patients at baseline

	Charnley	Spectron EF	Spectron EF
		Reflection	Reflection
	Ogee	CPE	XLPE
Item	(n = 30)	(n = 60)	(n = 60)
Female/male, n	20/10	43/17	42/18
Mean age (SD), years	70.0 (6.1)	69.1 (5.8)	70.1 (5.3)
Mean weight (SD), kg	76.0 (15)	73.9 (12.4)	77.9 (14.8)
Mean BMI (SD)	26.4 (3.9)	26.1 (3.5)	26.9 (4.0)
Primary/secondary			
osteoarthritis, n	28/2	52/8	49/11
Median cup size (range), mm	43 (40–43)	52 (49–61)	52 (43–58)
Mean follow up for the K–M			
analysis (SD), years	13.8 (4.1)	15.3 (2.9)	14.4 (4.1)

SD: standard deviation.

### Migration and wear at 2 years

At the 2-year follow-up, the mean proximal head penetration was 0.13 mm (95% confidence interval [CI] 0.10–15) for the Charnley group, 0.35 mm (CI 0.31–0.39) for the Reflection CPE group, and 0.08 mm (CI 0.06–0.11) for the Reflection XLPE group. There was no difference in bedding-in between the groups (Supplementary Table 1). The proximal migration at 2-year follow-up was 0.19 mm (CI 0.09–0.30) for the Charnley group, 0.07 mm (CI 0.02–0.11) for the Reflection CPE group, and 0.05 mm (CI 0.01–0.10) for the Reflection XLPE group (Supplementary Table 1).

### ROC curves

**Figure 4:**
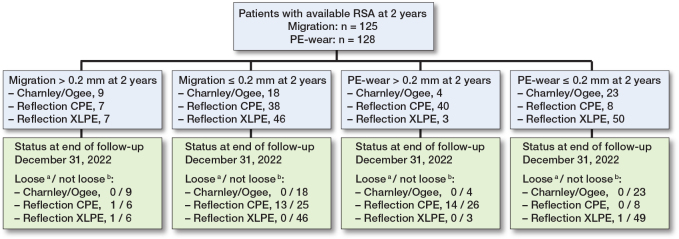
Flowchart showing the distribution of patients in categories below or over the thresholds for early migration and wear, and the number of implants within each category considered loose at the end of follow-up. **^a^** Considered as loose, either by revision for aseptic loosening, or by examination of latest available radiographs. **^b^** Considered as intact at end of follow-up or censored due to death or revision for causes other than aseptic loosening.

12 cups were revised for aseptic loosening of the cup alone or cup and stem combined, while 7 more cups were found to be loose when reviewing the latest available radiograph (Supplementary Table 2). Thus, a total of 19 cups, 17 CPE and 2 XLPE, were considered loose. For creation of ROC curves for prediction of loosening, when excluding the Charnley group, 98 patients had available RSA at 2 years for analysis of early migration and 101 patients had available RSA at 2 years for analysis of early PE wear. Among these patients 14 cups had migration over 0.2 mm while 43 had PE wear above 0.2 mm (Figure 4).

**Figure 5:**
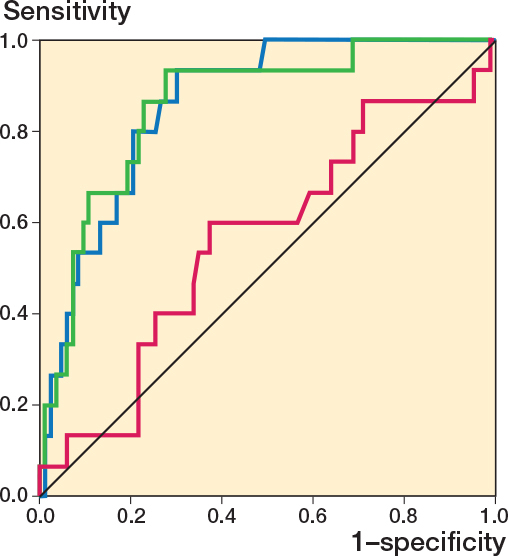
ROC curve for proximal cup migration (AUC 0.56, CI 0.40–0.73; purple) and PE wear (AUC 0.85, CI 0.77–0.94; blue) at 2 years. Green demonstrates the curve for both migration and PE wear simultaneously based on a logistic regression model. CI: 95% confidence interval.

**Figure 6:**
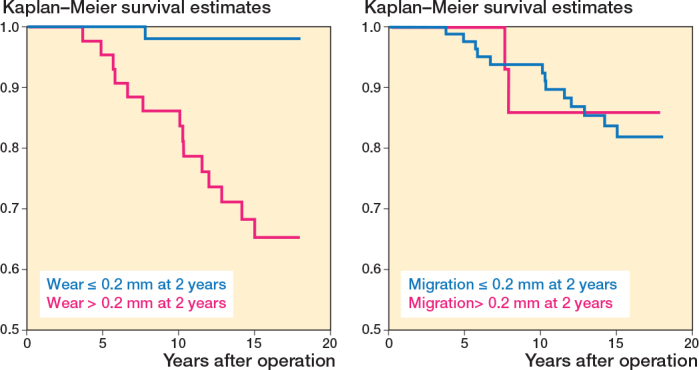
Kaplan–Meier survival curves for time to loosening stratified by migration (≤ 0.2 mm or > 0.2 mm) and wear (≤ 0.2 mm or > 0.2 mm) at 2-year RSA follow-up. The HR for migration over 0.2 mm was 0.88 (CI 0.20–3.89), while the HR for PE wear over 0.2 mm was 19.4 (CI 2.55–148). CI: 95% confidence interval.

The ROC curve for proximal cup migration from postoperatively to 2 years had an AUC of 0.56 (CI 0.40–0.73) (Figure 5). Migration of 0.2 mm at 2 years had a sensitivity of 0.13 and specificity of 0.86. For PE wear at 2-year follow-up the AUC was 0.85 (CI 0.77–0.94) (Figure 4). A wear rate of 0.1 mm/year (0.2 mm at 2 years) gave a sensitivity of 0.93 and specificity of 0.65. The difference in the AUC for early migration and wear was 0.29 (CI 0.09–0.49; P = 0.001). The HR for early migration over 0.2 mm was 0.88 (0.20–3.89), while the HR for early PE wear over 0.2 mm was 19.4 (CI 2.55–148) (Figure 6).

A ROC curve for migration between the 1- and 2-year follow-up had an AUC of 0.61 (CI 0.44–0.78) (Supplementary Figure 1).

All the loose CPE cups exhibited a wear rate of more than 0.1 mm/year (Supplementary Figure 2). Furthermore, 23 cups had proximal cup migration exceeding the risk of loosening threshold of 0.2 mm at 2 years. Of these cups, only 2 were ultimately found to be loose, with migration values of 0.3 and 0.7 mm, both involving CoCr heads and occurring within the XLPE and CPE groups respectively (Supplementary Table 2). None of the other cups identified as loose had migration values surpassing this threshold.

When including the Charnley group in the analyses, early migration had an AUC for the ROC curve of 0.52 (CI 0.36–0.67), with the sensitivity and specificity of the 0.2 mm threshold being 0.13 and 0.81 respectively. For early PE wear the AUC was 0.87 (CI 0.78–0.95), with sensitivity and specificity for wear rate of 0.1 mm/year being 0.93 and 0.67 respectively. The difference in AUC for the 2 ROC curves was 0.36 (CI 0.17–0.55; P < 0.001) (Supplementary Figure 3).

### Survival

The Kaplan–Meier analysis, with follow-up up to 18 years, showed that the CPE cups had an 18-year survival without aseptic cup loosening of 65% (CI 48–77), whereas the XLPE cups demonstrated a survival of 96% (CI 85–99) (Supplementary Figure 4). Unadjusted Cox analyses revealed a 9-fold higher (CI 2.1–38.9) risk of aseptic cup loosening for CPE cups compared with the XLPE cups. There were no instances of loosening observed for Charnley cups.

## Discussion

We aimed to investigate whether early wear or migration, measured via RSA, predicts later cup loosening. We also compared long-term aseptic loosening rates between CPE and XLPE cups.

We found that early PE wear served as a very good predictor for later loosening while early cup migration did not. Also, we showed less wear, aseptic loosening, and revision of XLPE cups compared with CPE.

A meta-analysis introduced specific thresholds for evaluating the risk of implant revision due to aseptic loosening based on the proximal migration at 2 years [3]. Implants with migration ranging between 0.2 and 1.0 mm were classified to be “at risk” of revision, while migration exceeding 1 mm was deemed as “unacceptable.” We found that all groups exhibited mean proximal migration of less than 0.2 mm at 2 years [10]. However, within this cohort, there were 23 individual cases classified as “at risk” at 2 years. Among these 23, only 2 ultimately experienced aseptic loosening.

One of the primary motivations for conducting RSA studies is the predictive value of early implant migration for later loosening [3,20-22]. However, this notion regards cup designs/brands as a group and not for the individual patient. This means that a design which exhibits higher mean migration values than another is more likely to have a higher incidence of loosening in the longer term. Including RSA in clinical practice has been discussed, especially considering newer RSA methods, such as CT-based RSA, which can be done without implanting markers [23]. As such, it is interesting as to whether thresholds can be found for migration and wear that could be applicable as diagnostic tests for the individual patient in the clinic. The AUC for the ROC curve created for proximal cup migration was 0.56. Also, the sensitivity of this 0.2 mm threshold was low. As reported, all groups had mean proximal migration of less than 0.2 mm at 2 years, and among the individual cases that were above this level only a few were revised. Thus, in our study early cup migration did not perform adequately as a prognostic test. At 2 years the Reflection cups had less migration than the Charnley cups. Both the Charnley cups and the Reflection All-Poly XLPE cups were stable from 2 years until the 10-year follow-up [12]. However, the Reflection All-Poly CPE cups continued to migrate from the 2-year follow-up, most notably in the hips with Oxinium heads [12]. No Charnley cups were ultimately found to be loose. The low levels of radiation (2.5 Mrad) used to sterilize the Charnley cup likely induced some degree of crosslinking in the polyethylene (PE), which may explain the enhanced durability and superior performance of this prosthesis compared with the Reflection All-Poly CPE cup, which lacked crosslinking [24]. In addition, the design difference with PMMA beads on the back of the Reflection cups might give instant stability and the Charnley flanged cup with no beads might give more initial movement, and later stability could contribute to higher early migration in the Charnley cups than the Reflection cups.

For wear the AUC was 0.85, which could be classified as a very good predictive ability, and the sensitivity and specificity were more acceptable. The RSA follow-up, which lasted up to 10 years, showed that the Reflection CPE cups had very high levels of wear [12]. After a while the previously fixed cups started to migrate at between 5 and 10 years, while the XLPE cups and Charnley cups remained stable. It is therefore reasonable to assume that the primary mechanism for aseptic loosening in the CPE cups was increased wear resulting in osteolysis and secondary migration [25].

It is possible that the high wear rate of the CPE cup was the dominating factor in the loosening mechanism of this cup, masking the effect of migration. The present study raises questions regarding the notion that RSA studies on migration with only 2 years’ follow-up are adequate. It is plausible that an implant is stable at 2 years, but starts to migrate or loosen after a longer period due to wear and bone resorption. In the XLPE groups there were 2 cups revised relatively early due to aseptic loosening, which may be due to poor cementation technique over osteolysis induced by PE particles for these 2 cups. Regarding the discussion between dose/response and wear rate as the explanation for wear-related loosening it is difficult to give support to either theory from this study. All loose cups, except for the one XLPE cup with RSA measurement, had a wear rate above 0.1 mm/year. However, with increasing wear rate there is also an increase in cumulative PE particle exposure. Therefore, this study does not give an unambiguous view in either direction.

It is difficult to establish the utility of these thresholds and our findings in clinical practice. First, as most acetabular cups now are made of XLPE, a wear rate of more than 0.1 mm/year is less likely. In addition, only 1 THA system for the creation of ROC and sensitivity and specificity was evaluated. Different findings could be possible with different designs, for instance in uncemented THA.

### Limitations

During the study period several patients were censored before the end of study, due to mortality or revision for causes other than aseptic loosening. In addition, some patients did not have available RSA measurements at 2 years. Furthermore, when the power analysis and execution of the study was done, the initial plan was for 2 years’ RSA follow-up, and the study design was not made explicitly for investigating the aims of this paper. It could therefore be underpowered in finding an effect for migration for loosening, for instance. It is possible that with a larger sample size, with only XLPE cups, the effect on loosening from early migration would become more evident. In addition, the threshold of 0.2 mm of proximal cup migration has been suggested for use in group-level analysis when investigating a specific implant, and not for individual patients, which was the application in this study. Some detection bias could also be possible, as the surgeons who carried out follow-up of the patients were aware of which implant the patient had. Therefore, if a cup were suspected to be loose, the surgeons might have had a lower threshold for revision if they knew that the patient had a poorly performing CPE cup.

A strength is that all patients were followed up until the end of study regarding revision surgery and death, as well as confirmation of loosening by investigating radiographs.

### Conclusion

We found that early PE wear served as a very good predictor for later loosening while early cup migration did not in cemented cups. Also, we showed less wear, aseptic loosening, and revision of XLPE cups compared with CPE.

### Supplementary data

Supplementary Tables 1–2 and Supplementary Figures 1–4 are available as supplementary data on the article page, doi: 10.2340/17453674.2025.44328

## Supplementary Material



## Notes

HGJ: data collection, analysis, and writing. GH, TK, OF: planning, data collection, and revision of the manuscript. AMF, AA, and KH: data collection, analysis, and revision of the manuscript. MB, BJ and KI: data collection and revision of the manuscript. SAL and SHLL: analysis and revision of the manuscript. PJH: revision of the manuscript.

Thanks are offered to Terje Stokke for providing all the radiographs, and to the surgeons who contributed to the study.

Handling co-editors: Bart A Swierstra and Robin Christensen

*Acta* thanks Dennis Janssen and Bart G Pijls for help with peer review of this manuscript.

## References

[1] Kärrholm J, Herberts P, Hultmark P, Malchau H, Nivbrant B, Thanner J. Radiostereometry of hip prostheses: review of methodology and clinical results. Clin Orthop Relat Res 1997; (344): 94-110. doi: 10.1097/00003086-199711000-00011. 9372762

[2] Madanat R, Mäkinen T J, Aro H T, Bragdon C, Malchau H. Adherence of hip and knee arthroplasty studies to RSA standardization guidelines: a systematic review. Acta Orthop 2014; 85(5): 447-55. doi: 10.3109/17453674.2014.934187. 24954489 PMC4164860

[3] Pijls B G, Nieuwenhuijse M J, Fiocco M, Plevier J W, Middeldorp S, Nelissen R G, et al. Early proximal migration of cups is associated with late revision in THA: a systematic review and meta-analysis of 26 RSA studies and 49 survival studies. Acta Orthop 2012; 83(6): 583-91. doi: 10.3109/17453674.2012.745353. 23126575 PMC3555453

[4] Callary S A, Solomon L B, Holubowycz O T, Campbell D G, Munn Z, Howie D W. Wear of highly crosslinked polyethylene acetabular components. Acta Orthop 2015; 86(2): 159-68. doi: 10.3109/17453674.2014.972890. 25301435 PMC4404765

[5] Dumbleton J H, Manley M T, Edidin A A. A literature review of the association between wear rate and osteolysis in total hip arthroplasty. J Arthroplasty 2002; 17(5): 649-61. doi: 10.1054/arth.2002.33664. 12168184

[6] Wilkinson J M, Hamer A J, Stockley I, Eastell R. Polyethylene wear rate and osteolysis: critical threshold versus continuous dose–response relationship. J Orthop Res 2005; 23(3): 520-5. doi: 10.1016/j.orthres.2004.11.005. 15885470

[7] Malak T T, Broomfield J A, Palmer A J, Hopewell S, Carr A, Brown C, et al. Surrogate markers of long-term outcome in primary total hip arthroplasty: a systematic review. Bone Joint Res 2016; 5(6): 206-14. doi: 10.1302/2046-3758.56.2000568. 27267795 PMC4921042

[8] Cho C H, Pijls B G, Abrahams J M, Roerink A, Katembwe R, Baker A, et al. Migration patterns of acetabular cups: a systematic review and meta-analysis of RSA studies. Acta Orthop 2023; 94: 626-34. doi: 10.2340/17453674.2023.24580. 38157007 PMC10757199

[9] Schulz K F, Altman D G, Moher D. CONSORT 2010 Statement: updated guidelines for reporting parallel group randomised trials. BMC Med 2010; 8: 18. doi: 10.1186/1741-7015-8-18. 20334633 PMC2860339

[10] Kadar T, Hallan G, Aamodt A, Indrekvam K, Badawy M, Skredderstuen A, et al. Wear and migration of highly cross-linked and conventional cemented polyethylene cups with cobalt chrome or Oxinium femoral heads: a randomized radiostereometric study of 150 patients. J Orthop Res 2011; 29(8): 1222-9. doi: 10.1002/jor.21389. 21360584

[11] Jonsson B A, Kadar T, Havelin L I, Haugan K, Espehaug B, Indrekvam K, et al. Oxinium modular femoral heads do not reduce polyethylene wear in cemented total hip arthroplasty at five years: a randomised trial of 120 hips using radiostereometric analysis. Bone Joint J 2015; 97-B(11): 1463-9. doi: 10.1302/0301-620x.97b11.36137. 26530646

[12] Johannessen H G, Hallan G, Kadar T, Fenstad A M, Lygre S H L, Haugan K, et al. Polyethylene wear and cup migration of cemented total hip arthroplasty with femoral heads made of oxidized zirconium, steel, or cobalt chromium: a 10-year secondary analysis from a randomized trial using radiostereometry. Acta Orthop 2024; 95: 578-85. doi: 10.2340/17453674.2024.41945. 39347798 PMC11441331

[13] Hardinge K. The direct lateral approach to the hip. J Bone Joint Surg Br 1982; 64(1): 17-19. doi: 10.1302/0301-620x.64b1.7068713. 7068713

[14] Valstar E R, Gill R, Ryd L, Flivik G, Börlin N, Kärrholm J. Guidelines for standardization of radiostereometry (RSA) of implants. Acta Orthopaedica 2005; 76(4): 563-72. doi: 10.1080/17453670510041574. 16195075

[15] Lydersen S. ROC-kurver og diagnostiske tester. Tidsskr Nor Laegeforen 2018; 138(15). doi: 10.4045/tidsskr.18.0542. 30277050

[16] Norwegian Arthroplasty Register Annual Report 2024. Available from: https://www.helse-bergen.no/48d1eb/contentassets/9f19d57711ee4e60815d6b89e8e8472b/report2024.pdf

[17] Stauffer R N. Ten-year follow-up study of total hip replacement. J Bone Joint Surg Am 1982; 64(7): 983-90. doi: 10.2106/00004623-198264070-00003. 7118986

[18] Hoo Z H, Candlish J, Teare D. What is an ROC curve? Emerg Med J 2017; 34(6): 357-9. doi: 10.1136/emermed-2017-206735. 28302644

[19] DeLong E R, DeLong D M, Clarke-Pearson D L. Comparing the areas under two or more correlated receiver operating characteristic curves: a nonparametric approach. Biometrics 1988; 44(3): 837-45. doi: 10.2307/2531595. 3203132

[20] Hauptfleisch J, Glyn-Jones S, Beard D J, Gill H S, Murray D W. The premature failure of the Charnley Elite-Plus stem: a confirmation of RSA predictions. J Bone Joint Surg Br 2006; 88(2): 179-83. doi: 10.1302/0301-620x.88b2.17055. 16434520

[21] Nieuwenhuijse M J, Valstar E R, Kaptein B L, Nelissen R G. The Exeter femoral stem continues to migrate during its first decade after implantation: 10–12 years of follow-up with radiostereometric analysis (RSA). Acta Orthop 2012; 83(2): 129-34. doi: 10.3109/17453674.2012.672093. 22401676 PMC3339525

[22] Kärrholm J, Borssén B, Löwenhielm G, Snorrason F. Does early micromotion of femoral stem prostheses matter? 4–7-year stereoradiographic follow-up of 84 cemented prostheses. J Bone Joint Surg Br 1994; 76(6): 912-17. doi: 10.1302/0301-620X.76B6.7983118. 7983118

[23] Kaptein B L, Pijls B, Koster L, Kärrholm J, Hull M, Niesen A, et al. Guideline for RSA and CT-RSA implant migration measurements: an update of standardizations and recommendations. Acta Orthop 2024; 95: 256-67. doi: 10.2340/17453674.2024.40709. 38819193 PMC11141406

[24] McKellop H, Shen F W, Lu B, Campbell P, Salovey R. Effect of sterilization method and other modifications on the wear resistance of acetabular cups made of ultra-high molecular weight polyethylene: a hip-simulator study. J Bone Joint Surg Am 2000; 82(12): 1708-25. doi: 10.2106/00004623-200012000-00004. 11130644

[25] Harris W H. Wear and periprosthetic osteolysis: the problem. Clin Orthop Relat Res 2001; (393): 66-70. doi: 10.1097/00003086-200112000-00007. 11764372

