# Supplementary material for: Early cup migration and wear as predictors for later aseptic loosening: a secondary evaluation of a randomized controlled RSA trial on cemented hip arthroplasties with 18-year follow-up
Source: Acta Orthop. 2025 Aug 15;96:618–24. doi: 10.2340/17453674.2025.44328 (PMC12357177; doi:10.2340/17453674.2025.44328)
Supplement: Supplementary file 1 [file ActaO-96-44328-s1.pdf]

Supplementary data

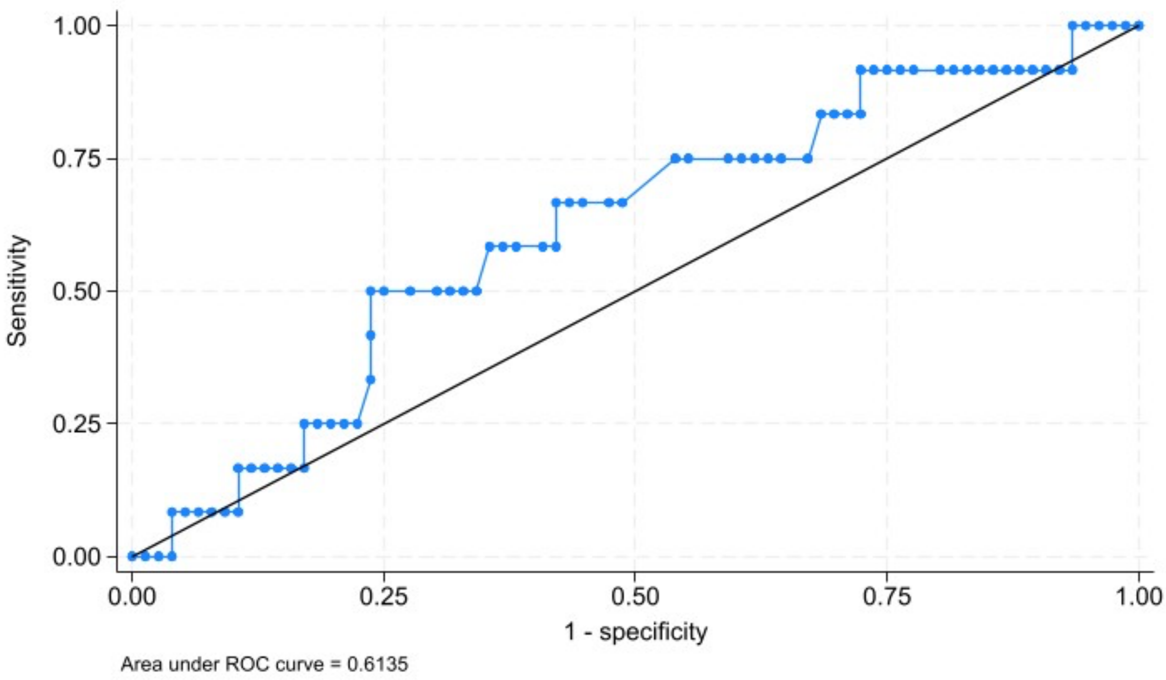

Supplementary Figure 1. ROC-curve for migration between 1- and 2-year follow-up.

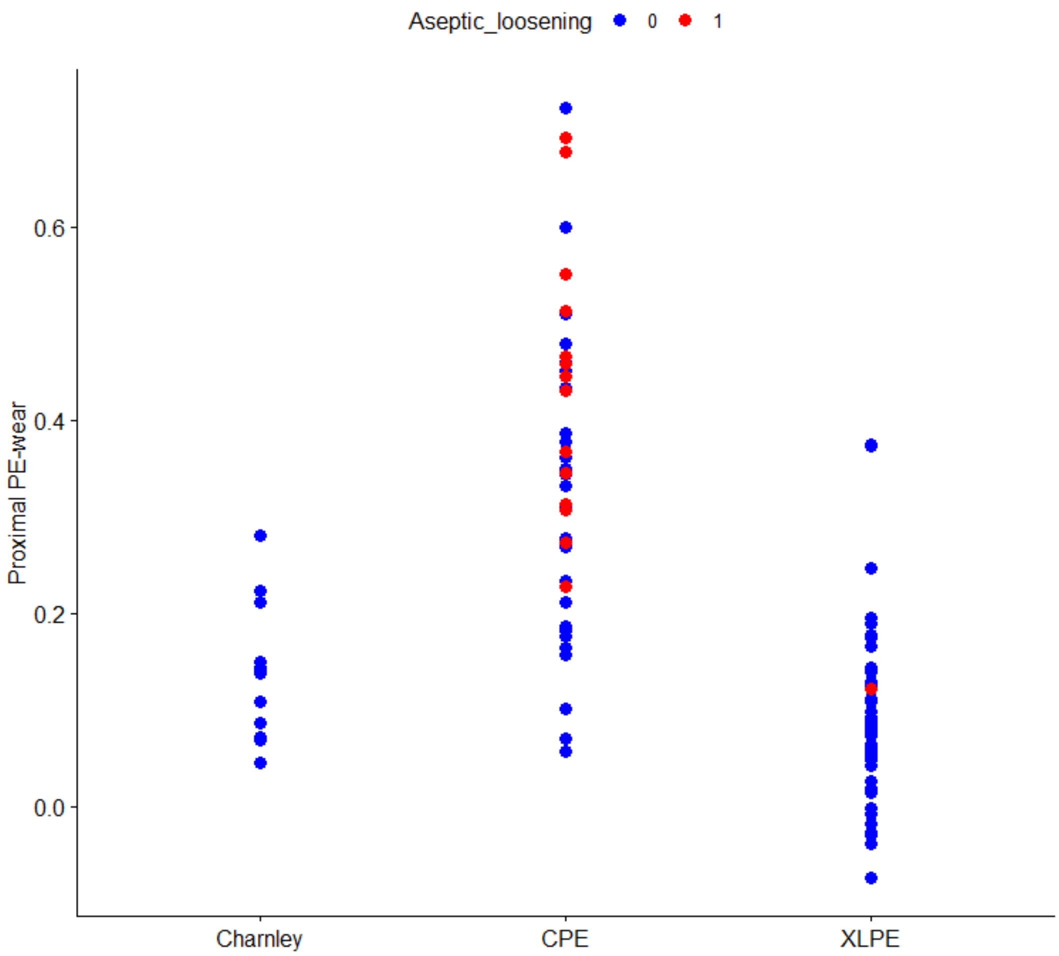

Supplementary Figure 2. Cemented total hip arthroplasty with 3 different groups. Scatterplot showing the spread of proximal head penetration at 2 years of all hips with available RSA measurements for the 3 groups. The red dots represent the hips that were revised due to aseptic loosening or were found to be radiographically loose.

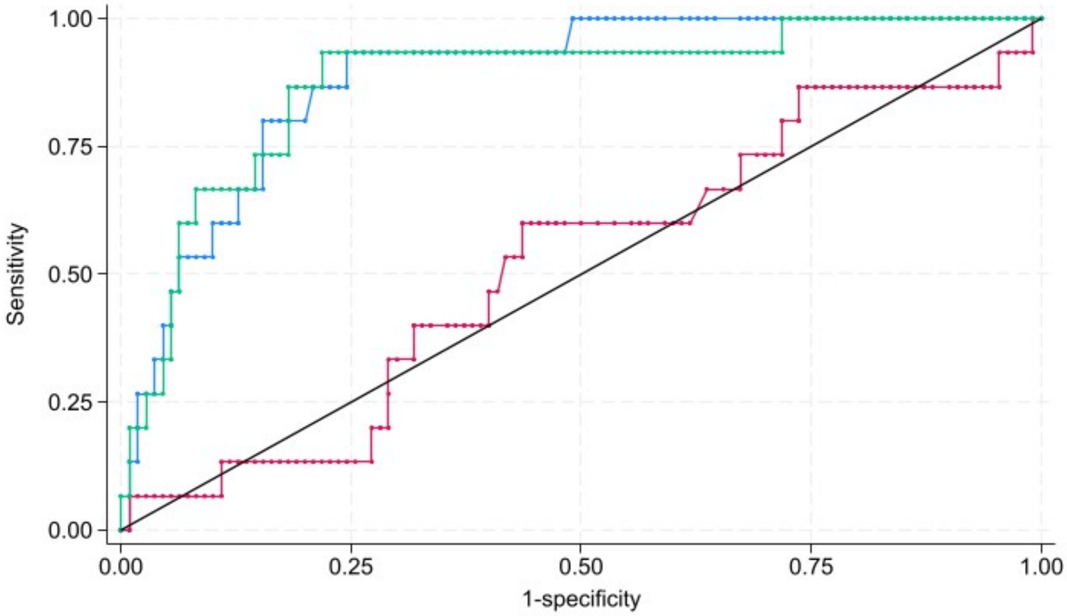

Supplementary Figure 3. ROC curve for proximal cup migration (AUC 0.52, CI 0.36–0.67; purple) and PE-wear (AUC: 0.87, CI 0.78–0.95; blue) at 2 years. Green demonstrates the curve for both migration and PE-wear simultaneously based on a logistic regression model including the Charnley group.

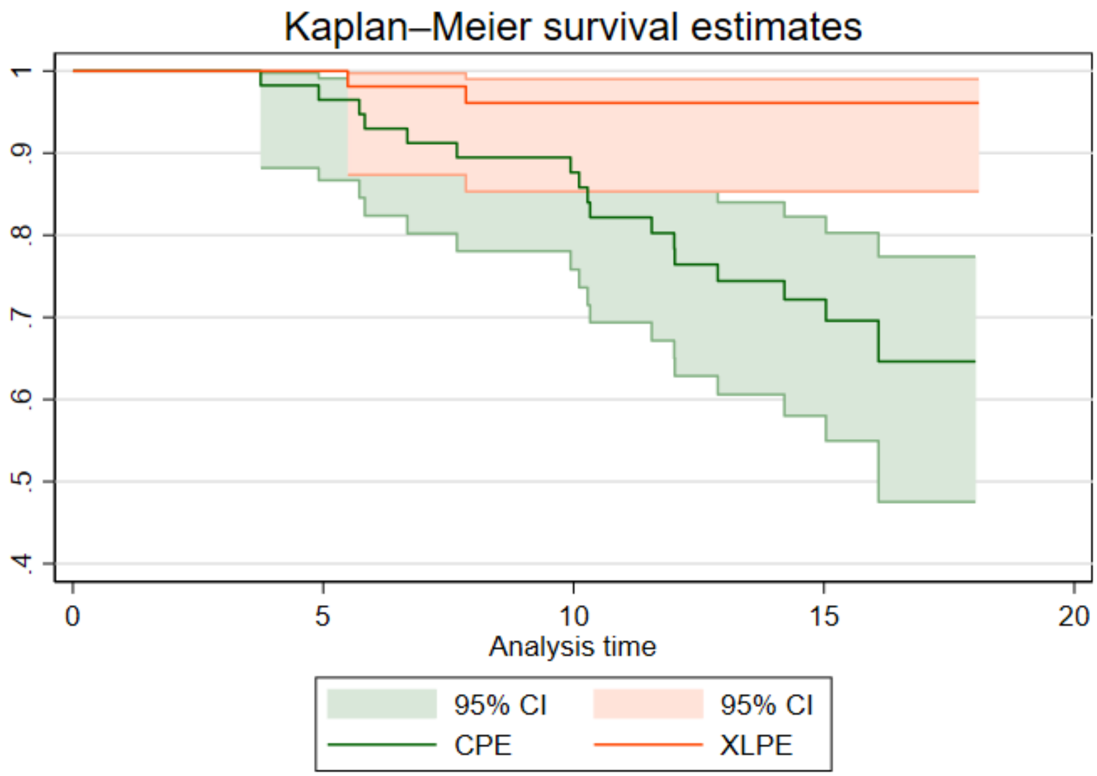

Supplementary Figure 4. Cemented total hip arthroplasty with Reflection All Poly CPE vs XLPE cups. Kaplan-Meier-curve showing the survival of the conventional polyethylene (CPE) cups, with CoCr and OxZr heads, and XLPE cups with CoCr and OxZr heads with endpoint aseptic loosening (revised for loosening or radiologically loose).

Supplementary Table 1. RSA results at 2 years follow-up, used to create ROC-curves for the risk of later loosening

	Charnley/Ogee	Spectron EF/ Reflection CPE	Spectron EF/ Reflection XLPE
Total head penetration [a] (CI), mm			
3 months	0.05 (0.03–0.07) (n = 26)	0.05 (0.02–0.07) (n = 39)	0.04 (0.02–0.06) (n = 48)
6 months	0.07 (0.05–0.09) (n = 26)	0.09 (0.07–0.12) (n = 40)	0.06 (0.04–0.09) (n = 45)
1 year	0.09 (0.07–0.10) (n = 28)	0.18 (0.14–0.21) (n = 39)	0.06 (0.04–0.08) (n = 51)
2 years	0.13 (0.10–0.15) (n = 26)	0.35 (0.31–0.39) (n = 48)	0.08 (0.06–0.11) (n = 53)
Bedding-in [b] (CI), mm	0.05 (0.03–0.07) (n = 26)	0.05 (0.02–0.07) (n = 39)	0.04 (0.02–0.06) (n = 48)
Wear from 3-month–2-year follow-up (CI), mm	0.08 (0.05–0.11) (n = 25)	0.29 (0.24–0.34) (n = 38)	0.05 (0.03–0.06) (n = 48)
Wear rate [c] (CI), mm/year	0.05 (0.03–0.06) (n = 25)	0.17 (0.14–0.20) (n = 38)	0.03 (0.02–0.04) (n = 48)
Proximal migration at 2 years (CI), mm)	0.19 (0.09–0.30) (n = 27)	0.07 (0.02–0.11) (n = 45)	0.05 (0.01–0.10) (n = 53)

[a] Total head penetration from post-operatively until the given time point.

[b] Head penetration from postoperatively to 3-months follow-up

[c] Wear rate, calculated as annual wear rate from 1-year to 2-year follow-up

Supplementary Table 2. Cemented total hip arthroplasty with 3 different groups. List of radiologically loose (top half) and surgically revised patients (bottom half) during the study period with reason and time to revision/loosening. The acetabular liner wear and migration along the y-axis at 2 years are included. (-) denotes missing measurements

Study No.	Radiological loose cups (not revised)	Time to loosening	Group [a]	Wear at 2 years (postop. to 2-year follow up), mm	Y-translation at 2 years, mm	Y-rotation at 2 years, degree
1		138 months 23 days	2	0.55	0.01	0.51
2		144 months 7 days	2	0.37	0.003	0.85
3		180 months 16 days	2	0.27	–0.02	0.05
4		70 months	2	0.23	–0.01	0.99
5		81 months	2	0.31	0.1	–0.21
6		121 months 12 days	2	0.47	0.07	–0.36
7		193 months 3 days	2	-	-	-
	**Surgically revised (reason for revision)**	**Time to revision**	**Group**	**Femoral head penetration at 2 years**	**Y-translation at 2 years, mm**	**Y-rotation at 2 years, degree**
1	Loose cup	80 months 27 days	2	0.68	–0.11	0.26
2	Loose cup and stem	94 months 23 days	2	0.69	0.65	–0.15
3	Loose cup	120 months 5 days	2	-	-	-
4	Loose cup and stem	178 months 4 days	2	0.35	–0.22	0.53
5	Loose cup	201 months 19 days	2	0.46	0.15	–1.61
6	Loose cup	72 months 6 days	2	0.31	0.05	0.64
7	Loose cup	97 months 9 days	2	0.51	0.11	0.46
8	Loose cup and stem	125 months 6 days	2	0.43	0.06	0.39
9	Loose cup	150 months 15 days	2	-	-	-
10	Loose cup and stem	163 months 3 days	2	0.45	0.13	0.35
11	Loose cup and stem	98 months 27 days	3	0.12	0.31	–1.07
12	Loose cup	66 months 11 days	3	-	-	-
13	Dislocation	61 months 28 days	1	0.21	0.06	0.27
14	Infection	151 months 28 days	2	0.16	0.17	–0.49
15	Infection	17 days	2	-	-	-
16	Infection	21 days	2	-	-	-
17	Infection	24 months 8 days	3	-	-	-
18	Infection	20 days	3	-	-	-
19	Infection	41 months 19 days	3	0.09	–0.1	–0.01

[a] See Figure 2 for group definitions
